# *ATP6V0C* Is Associated With Febrile Seizures and Epilepsy With Febrile Seizures Plus

**DOI:** 10.3389/fnmol.2022.889534

**Published:** 2022-05-06

**Authors:** Yang Tian, Qiong-Xiang Zhai, Xiao-Jing Li, Zhen Shi, Chuan-Fang Cheng, Cui-Xia Fan, Bin Tang, Ying Zhang, Yun-Yan He, Wen-Bin Li, Sheng Luo, Chi Hou, Wen-Xiong Chen, Wei-Ping Liao, Jie Wang

**Affiliations:** ^1^Department of Neurology, Guangzhou Women and Children’s Medical Center, Guangzhou Medical University, Guangzhou, China; ^2^Department of Pediatrics, Guangdong Provincial People’s Hospital, Guangdong Academy of Medical Sciences, Guangzhou, China; ^3^Department of Neurology, Institute of Neuroscience, The Second Affiliated Hospital of Guangzhou Medical University, Guangzhou, China; ^4^Key Laboratory of Neurogenetics and Channelopathies of Guangdong Province, Ministry of Education of China, Guangzhou, China; ^5^The Seventh Affiliated Hospital, Sun Yat-sen University, Shenzhen, China

**Keywords:** *ATP6V0C*, loss of function, febrile seizures, epilepsy with febrile seizures plus, whole-exome sequencing

## Abstract

**Purpose:**

To identify novel genetic causes of febrile seizures (FS) and epilepsy with febrile seizures plus (EFS+).

**Methods:**

We performed whole-exome sequencing in a cohort of 32 families, in which at least two individuals were affected by FS or EFS+. The probands, their parents, and available family members were recruited to ascertain whether the genetic variants were co-segregation. Genes with repetitively identified variants with segregations were selected for further studies to define the gene-disease association.

**Results:**

We identified two heterozygous *ATP6V0C* mutations (c.64G > A/p.Ala22Thr and c.361_373del/p.Thr121Profs*7) in two unrelated families with six individuals affected by FS or EFS+. The missense mutation was located in the proteolipid c-ring that cooperated with a-subunit forming the hemichannel for proton transferring. It also affected the hydrogen bonds with surround residues and the protein stability, implying a damaging effect. The frameshift mutation resulted in a loss of function by yielding a premature termination of 28 residues at the C-terminus of the protein. The frequencies of *ATP6V0C* mutations identified in this cohort were significantly higher than that in the control populations. All the six affected individuals suffered from their first FS at the age of 7–8 months. The two probands later manifested afebrile seizures including myoclonic seizures that responded well to lamotrigine. They all displayed favorable outcomes without intellectual or developmental abnormalities, although afebrile seizures or frequent seizures occurred.

**Conclusion:**

This study suggests that *ATP6V0C* is potentially a candidate pathogenic gene of FS and EFS+. Screening for *ATP6V0C* mutations would help differentiating patients with Dravet syndrome caused by *SCN1A* mutations, which presented similar clinical manifestation but different responses to antiepileptic treatment.

## Introduction

*ATP6V0C* (OMIM*108745), located at chromosomal locus 16p13.3 and spans approximately 6 kb of genomic DNA, is highly expressed in the brain, predominantly in the brain cortex across whole lifespan.^1^ It encodes a 155-amino acid H+ transport protein ATP6V0C that widely distributed in lysosomes, endosomes, Golgi-derived vesicles, secretory vesicles, and plasma membrane for some cell types (Toei et al., 2010). ATP6V0C is the c-subunit of the V0 domain of vacuolar ATPase (V-ATPase) that primarily mediates acidification of eukaryotic intracellular organelles and is necessary for numerous intracellular processes including protein sorting, zymogen activation, receptor-mediated endocytosis, and synaptic vesicle proton gradient generation (Morel, 2003). As a component of V0 domain of V-ATPase, ATP6V0C forms the proteolipid c-ring of the V-ATPase and plays a pivotal role in synaptic vesicle proton gradient generation and regulation of intra and extracellular *pH* value (Higashida et al., 2017; Xu et al., 2019). Experiments in zebrafish ortholog revealed that *ATP6V0C2* is associated with neurotransmitter storage/secretion and involved in the control of neuronal excitability (Chung et al., 2010). Homozygous *ATP6V0C* knockout mice exhibit neonatal lethality, abnormal embryonic tissue morphology, and failure of zygotic cell division.^2^ However, the relationship between *ATP6V0C* variants and human disease remains to be explored.

Febrile seizures (FS) are the most frequent convulsion events in childhood and represent the majority of childhood seizures with an incidence as high as 4–10% (Hackett et al., 1997; Gupta, 2016; Smith et al., 2019). These patients have five to seven times higher risk of developing subsequent afebrile seizures (epilepsy with febrile seizures plus, EFS+), compared to the general population (Annegers et al., 1987; Hedera et al., 2006). Etiologically, FS and EFS+ cases are usually cryptogenic without any secondary causes, implying potential associations with genetic factors. Several genes have been reported to be associated with FS or EFS+, including *ADGRV1*, *CHD2*, *CPA6*, *DEPDC5*, *DYRK1A*, *FGF13*, *GABRA1*, *GABRB3*, *GABRD*, *GABRG2*, *HCN1*, *HCN2*, *IMPA2*, *NPRL3*, *PCDH19*, *PRRT2*, *SCN1A*, *SCN1B*, *SCN2A*, *SCN9A*, *SLC12A5*, *SLC32A1*, *SRP9*, *STX1B*, *STXBP1*, and *YWHAG* (OMIM^3^) (Carvill et al., 2014; Rigbye et al., 2016; Deng et al., 2018; Liu et al., 2020; Heron et al., 2021; Ye et al., 2021). However, the genetic causes in most of the patients with FS or EFS+ remain to be elucidated.

In this study, we employed a whole-exome sequencing approach for discovery novel genetic causes in a cohort of familial cases with FS or EFS+. We identified two heterozygous *ATP6V0C* mutations that co-segregated with the disease in two unrelated families with six individuals affected.

## Materials and Methods

### Patients

A cohort of 32 families, in which at least two individuals were affected by FS or EFS+, were recruited. The patients were from the Departments of Neurology, Guangzhou Women and Children’s Medical Center and Guangdong Provincial People’s Hospital since June 2018 to April 2021.

Clinical data of the affected individuals were comprehensively collected, including present age, gender, age at seizure onset, seizure course, complete family history, response to antiseizure medications, and general and neurological examination results. Brain Magnetic resonance imaging (MRI) scans were performed to exclude abnormalities in brain structure. Video-electroencephalography (EEG) monitoring records, including hyperventilation, intermittent photic stimulation, open-close eyes test, and sleeping recording, were collected and reviewed by two qualified investigators. Epileptic seizures and epilepsy syndromes were diagnosed and classified according to Commission on Classification and Terminology of the Commission on Classification and Terminology of the International League Against Epilepsy (2010, 1989), Engel and International League Against Epilepsy (ILAE) (2001), Berg et al. (2010), and Scheffer et al. (2017). FS and EFS+ was diagnosed as that previously study described (Ye et al., 2021). The patients, their parents, and other affected family members were screened for genetic variants by the whole-exome sequencing. A cohort of 296 healthy Chinese volunteers were recruited as a control group. This study was approved by the ethics committee of Guangzhou Women and Children’s Medical Center and the ethics committee of Guangdong General Hospital. Written informed consent was obtained from the patient’s guardian.

### Whole-Exome Sequencing and Genetic Analysis

Whole blood samples were collected from the probands, their parents, and available family members to ascertain whether the genetic variants were co-segregation. Qiagen Flexi Gene DNA kit (Qiagen, Hilden, Germany) was used to extract genomic DNA from the whole blood samples. Whole-exome sequencing was performed on an Illumina HiSeq 2000 system by Beijing Genomics Institute (BGI) (Shenzhen, China) as previously described (Wang et al., 2018, 2021; Shi et al., 2019). The sequencing data were generated by massive parallel sequencing with more than 125 times average depth and more than 98% coverage in the capture region of the chip, which fulfills the high-quality criteria. Then, the raw data were aligned to the Genome Reference Consortium Human Genome build 37 (GRCh37) by Burrows–Wheeler alignment. Single-nucleotide point variants and indels were called with the Genome Analysis Toolkit. To obtain the comprehensive list of candidate pathogenic variants in each family, we adopted a case-by-case analytical pattern. We first removed the common variants with a minor allele frequency > 0.005 in Genome Aggregation Database (gnomAD). We then prioritized functional variants (protein-altering) including frameshift, nonsense, canonical splice site, initiation codon, and missense variants predicted as being damaging or probably damaging by *in silico* tools.^4^ Finally, we applied a co-segregation analysis model to screen for possibly disease-causing variants in each family. To identify novel epilepsy-associated gene, we put the known epilepsy-associated genes aside. Genes with repetitively identified variants with segregations were selected for further studies to define the gene-disease association. *ATP6V0C* appeared as one of the candidate genes with recurrent variants in this cohort. PCR-Sanger sequencing was performed to validate the candidate pathogenic variants on ABI 3730 sequencer (Applied Biosystems, Foster City, CA, United States). All *ATP6V0C* variants identified in this study were annotated to reference transcript NM_001694.

### Molecular Structural Analysis

To explore the effect of the *ATP6V0C* missense variant on protein structure, we performed protein models using Phyre2^5^ and AlphaFold.^6^ The three-dimensional (3D) structures of protein model were analyzed and visualized by PyMOL 2.3.^7^ In addition, we applied I-Mutant 3.0 program to predict the effect of the missense variant on protein stability.^8^ The alteration of the protein stability was assessed by the free energy change value (DDG, Kcal/mol). Negative DDG value means that the mutated protein possesses less stability and vice versa.

### Statistical Analysis

An aggregate analysis for *ATP6V0C* variants was performed in GraphPad Prism 7.00. The two-tailed Fisher’s exact test was applied to compare the frequency of *ATP6V0C* variants in this cohort with that in the control populations of gnomAD and our 296 normal control subjects. The cutoff value for statistical significance is 0.05.

## Results

### Identification of *ATP6V0C* Variants

**FIGURE 1:**
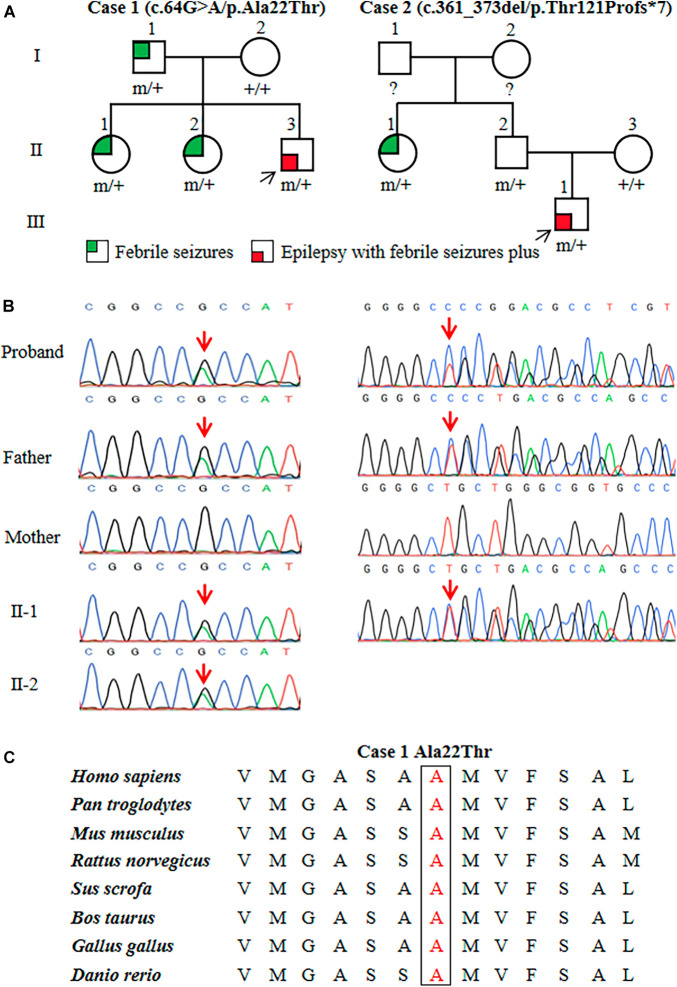
Genetic data of cases with *ATP6V0C* mutations. **(A)** Pedigrees of the two families with *ATP6V0C* mutations and their corresponding phenotypes. Individuals with mutation are marked as m/+, and those without mutation are marked as +/+. **(B)** DNA sequencing chromatograms of the two families with *ATP6V0C* mutations. Red arrows indicate the positions of the mutations. **(C)** Amino acid sequence alignment of the missense mutation shows that residue Ala22 is highly conserved in various species.

Two heterozygous variants in the *ATP6V0C* gene (OMIM*108745) were identified in two unrelated families with FS or EFS+, including one missense variant (c.64G > A/p.Ala22Thr) and one frameshift variant (c.361_373del/p.Thr121Profs*7) (Figures 1A,B). The c.64G > A/p.Ala22Thr variant was identified in a family with four affected individuals, in which the variant co-segregated with the phenotypes. The c.361_373del/p.Thr121Profs*7 variant was detected in family 2 with two affected individuals. The missense variant (c.64G > A/p.Ala22Thr) was predicted to be damaging or probably damaging by 14 out of 21 commonly used *in silico* prediction tools (Supplementary Table 1). The amino acid sequence alignment indicated that the Ala22Thr variant was located at a residue that is highly conserved across various species (Figure 1C). The c.361_373del/p.Thr121Profs*7 variant was considered as potentially pathogenic, which introduced a translational frameshift transcript that gave rise to a non-functional ATP6V0C protein or haploinsufficiency.

The two *ATP6V0C* variants were not observed in any public databases (including 1000 Genomes Project, Exome Sequencing Project, and gnomAD), neither in our 296 normal control subjects. Statistical analyses were performed to compare the frequencies of *ATP6V0C* variants in this cohort and control populations of gnomAD and the 296 normal subjects. Two mutant alleles in a total of 64 alleles (32 cases) were identified in this cohort. Statistically significant differences were observed in aggregate frequencies of the mutant alleles between this cohort and the controls of gnomAD-East Asian, gnomAD-all population, and our 296-normal control cohort (2/64 vs. 0/19750, *p* = 1.03 × 10^–5^; 2/64 vs. 0/280788, *p* = 5.11 × 10^–8^; 2/64 vs. 0/592, *p* = 9.38 × 10^–3^, respectively) (Table 1).

**TABLE 1:** Analysis of the aggregate frequency of *ATP6V0C* variants identified in this study.

	Allele count/number in this study (%)	Allele count/number in gnomAD-all populations (%)	Allele count/number in gnomAD-East Asian population (%)	Allele count/number in 296 normal controls of Chinese population (%)
**Identified *ATP6V0C* mutation**				
c.64G > A/p.Ala22Thr	1/64 (1.56)	−/−	−/−	−/−
c.361_373del/p.Thr121Profs*7	1/64 (1.56)	−/−	−/−	−/−
Total	2/64 (3.12)	0/280788	0/19750	0/592
*P* value^†^		5.11 × 10^–8^	1.03 × 10^–5^	9.38 × 10^–3^
OR (95% CI)		Inf (840.15-Inf)	Inf (58.51-Inf)	Inf (1.75-Inf)

*^†^P values and odds ratio were estimated with a 2-sided Fisher’s exact test.*

*CI, confidence interval; gnomAD, Genome Aggregation Database; OR, odds ratio.*

In the two families, we did not identify pathogenic or likely pathogenic variants in the 977 genes known to be associated with epileptic seizures (Wang et al., 2017).

### Effect of the Missense Mutation on Molecular Structure

**FIGURE 2:**
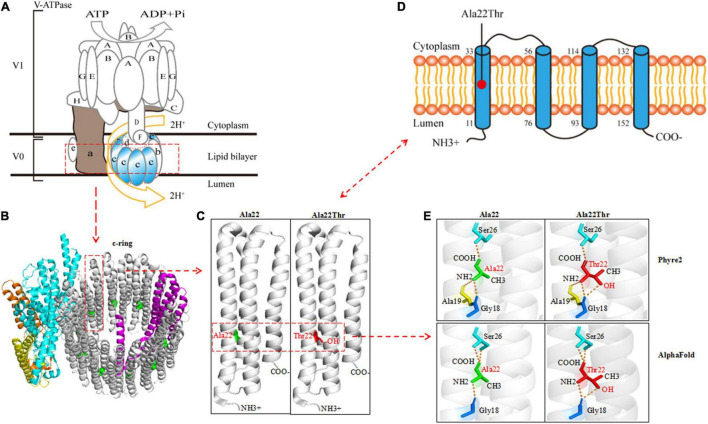
Schematic illustration of the ATP6V0C protein and molecular effect of Ala22Thr on ATP6V0C protein. **(A)** Structure diagram of major molecular components of vacuolar ATPase (V-ATPase), which is composed of a cytosolic V1 domain and a transmembrane V0 domain. The V0 domain consists of five different subunits: a, b, c, d, and e. The proteolipid c-ring was comprised of nine c-subunits (ATP6V0C protein) and one b-subunit. The “c-ring” couples the energy generated by ATP hydrolysis to the translocation of protons from the cytosol to the lumen through the hemichannel formed between the a-subunit and the proteolipid c-ring. **(B)** Three-dimensional (3D) structure of the V0 domain. **(C)** The focal structure of ATP6V0C protein. Ala22Thr was located in the first transmembrane domain of ATP6V0C protein with an addition of hydroxide radical. **(D)** The commonly used topographical diagram showing the five topological domains and the four transmembrane domains. **(E)** Left: residue Ala22 formed three hydrogen bonds with Gly18, Ala19, and Ser26 in Phyre2; AlphaFold showed that residue Ala22 formed three hydrogen bonds, one with Gly18 and two with Ser26. Right: both predictions showed that a novel hydrogen bond with Gly18 was formed when alanine at residue Ala22 was substituted by threonine. Furthermore, the Ala22Thr lead to a new addition of hydroxide radical that potentially influence the proton transport in the proteolipid c-ring of the hemichannel.

ATP6V0C protein is the c-subunit of the V0 domain of V-ATPase that is composed of a cytosolic V1 domain and a transmembrane V0 domain and functions as an ATP-dependent proton pump (Higashida et al., 2017; Xu et al., 2019; Wang et al., 2020). The proteolipid c-ring that cooperated with a-subunit forming the hemichannel for proton transfer was comprised of nine c-subunits and one b-subunit (Figures 2A,B). The c-subunit, also known as V-ATPase 16 kDa proteolipid subunit and encoded by *ATP6V0C*, is composed of five topological domains and four transmembrane domains^9^ (Figures 2C,D). Three topological domains (1–10, 77–92, and 153–155) are located within the lumen. The remaining two topological domains (34–55 and 115–131) are located within the cytoplasm. Four helical domains (11–33, 56–76, 93–114, and 132–152) are transmembrane. The Ala22Thr mutation was located in the first transmembrane segment of ATP6V0C protein (Figures 2C,D). The three-dimensional (3D) structure of the c-subunit was performed by Phyre2 and PyMOL (Figures 2B,C and Supplementary Figure 1). Considering that the Ala22Thr mutation was a heterozygote, so there should be at least four Ala22Thr mutants located in the proteolipid c-ring of V-ATPase. The molecular effect caused by the Ala22Thr mutation was further analyzed using available templates in Phyre2 and AlphaFold (Figure 2E). Residue Ala22 formed three hydrogen bonds with Gly18, Ala19, and Ser26 (Phyre2). AlphaFold showed that residue Ala22 formed three hydrogen bonds, one with Gly18 and two with Ser26. Both predictions showed that a novel hydrogen bond with Gly18 was formed when alanine at residue Ala22 was substituted by threonine (Figure 2E). Furthermore, the Ala22Thr lead to a new addition of hydroxide radical that potentially influence the proton transport in the hemichannel formed by proteolipid c-ring and a-subunit (Figures 2C,E). In addition, I-Mutant 3.0 program showed that the Ala22Thr cause a large decrease of the protein stability with a negative DDG value of 0.76 Kcal/mol.

### Clinical Features

*ATP6V0C* mutations were identified in two families with six affected individuals (Figures 1A,B, and Table 2). All affected individuals presented FS. The major clinical characteristics of the six patients were listed in Table 2.

**TABLE 2:** Clinical features of individuals with *ATP6V0C* mutations.

Cases	Sex	Age	Seizure onset	Seizure course	Seizure-free duration	AEDs	EEG	Brain MRI	Development	Diagnosis
**Case 1 (c.64G>A/p.Ala22Thr)**										
I-1	M	44 yr	8 mo	GTCS (FS), 1–2 times/yr for 5 yr	38 yr	No	NA	NA	Normal	FS
II-1	F	14 yr	8 mo	GTCS (FS), 1–2 times/yr for 5 yr	8 yr	No	Normal	Normal	Normal	FS
II-2	F	12 yr	8 mo	GTCS (FS), 1–2 times/yr for 5 yr	6 yr	No	Normal	Normal	Normal	FS
II-3	M	5 yr	8 mo	GTCS (FS), 5–6 times/yr for 1.5 yr; myoclonic seizures, up to 1–2 times/d for 1.5 yr	1 yr	VPA, NZP, LTG	Ictal: generalized PSW; interictal: generalized 2^~^4 Hz SW, irregular SSW and PSW	Normal	Normal	EFS+
Case 2 (c.361_373del/p.Thr121Profs*7)										
II-1	F	30 yr	8 mo	GTCS (FS), 0–2 times/yr for 4 yr	25 yr	No	Normal	NA	Normal	FS
III-1	M	3 yr	7 mo	GTCS (FS or aFS), 6–8 times/yr in cluster for 1.5 yr	1 yr	VPA	Generalized 2.5^~^3.5 Hz SW	Normal	Normal	EFS+

*AEDs, antiepileptic drugs; aFS, afebrile seizure; d, day; EEG, electroencephalogram; EFS+, epilepsy with febrile seizure plus; F, female; FS, febrile seizure; GTCS, generalized tonic–clonic seizure; LTG, lamotrigine; M, male; mo, months; MRI, magnetic resonance imaging; NA, not available; NZP, nitrazepam; PSW, polyspike-slow waves; SSW, spike-slow waves; SW, slow activities; VPA, valproate; wk, week; yr, years.*

**FIGURE 3:**
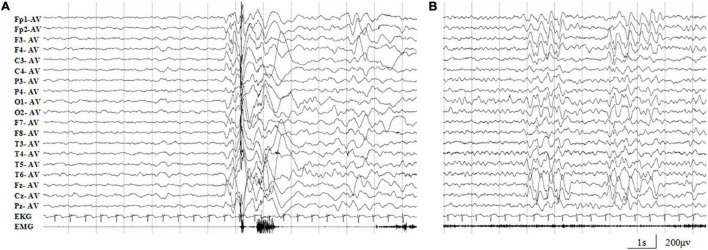
Representative EEG recordings of the case 1. **(A)** Ictal EEG showed high-amplitude generalized polyspike-slow waves associated with myoclonic seizures. **(B)** Interictal paroxysmal generalized 2∼4 Hz slow activity, or irregular spike-slow waves with anterior predominance during wakefulness.

The missense mutation (c.64G > A/p.Ala22Thr) was identified in family 1 with four individuals affected. The proband was a 5-year-old boy who had his first seizure at the age of 8 months with a fever of 38.5°C. It was a GTCS and lasted for 2–3 min. The febrile GTCS occurred approximately once every 2 months and tended to occur at a lower temperature (even at 37.3°C) later. Subsequently, afebrile GTCS occurred at the age of 1.5 years. Valproate (VPA) was then administered at a dose of 20 mg/kg/day. The seizure frequency decreased to around once every 4 months. At the age of 2 years and 2 months, the patient started to have myoclonic seizure with a frequency up to 1–2 times per day. Nitrazepam (NZP) was started (0.5 mg/kg/day) and the seizure frequency became 1 time per month within 4 months. At the age of 3 years and 8 months, lamotrigine (LTG) was added with a decrease of NZP, and the patient became seizure-free with VPA of 24.2 mg/kg/day, NZP of 0.3 mg/kg/day, and LTG of 1.2 mg/kg/day. Ictal video EEG recordings showed generalized polyspike-slow waves associated with myoclonic seizures (Figure 3A). Interictally, paroxysmal generalized 2∼4 Hz slow activities and irregular spike-slow waves (Figure 3B), or polyspike-slow waves with anterior predominance were recorded during wakefulness. The patient was born to non-consanguineous Chinese parents after a normal pregnancy and delivery. No positive signs in general and neurologic examinations were found. His intelligence, development, and growth were normal. His brain MRI scan, blood and cerebrospinal fluid routine biochemistry, metabolic, and pathogenic tests were normal. Three other members in this family, the father and two elder sisters, were affected by FS. They all had same seizure onset age and course from 8 months to 5 years old, normal intellectual and physical developments, and became seizure-free without antiepileptic therapy.

The proband in the family with Thr121Profs*7 was a 3-year-old boy who experienced FS at the age of 7 months. The FS then occurred every month in cluster, which consisted of 2–3 seizures. At the age of 1 year and 2 months, the patient suffered from afebrile GTCS. Valproate was then given at a dose of 21.2 mg/kg/day, and the seizure frequency reduced to once every 3 months. The patient became seizure-free with VPA of 23 mg/kg/day. The patient was born to non-consanguineous Chinese parents after a full-term pregnancy. He had an appropriate early developmental and had no physical malformations. His brain MRI and blood routine biochemical examination were normal. Video-EEG showed interictal paroxysmal generalized 2.5∼3.5 Hz slow activity. His paternal aunt carried the same mutation and was affected by FS in childhood. She had infrequent FS from 8 months to 4 years old and then became seizure free without medications. Both the proband and his paternal aunt had normal intellectual and physical developments.

## Discussion

The *ATP6V0C* gene is ubiquitously expressed across the whole lifespan and highly expressed in the central nervous system, prominently in the brain cortex, cerebellum, hypothalamus, and hippocampus (see text footnote 1). It encodes a 155-amino acid H+ transport protein ATP6V0C that is the c-subunit of the V0 domain of V-ATPase. V-ATPase is composed of a peripheral V1 domain that consists of A, B, C, D, E, F, G, and H subunits, and an integral V0 domain that consists of five different subunits: a, b, c, d, and e (Figures 2A,B). As one component of the multi-subunit enzyme complex of V-ATPase, ATP6V0C forms the proteolipid c-ring of the V-ATPase (Figures 2A,B), which is critical for the transport of H+ flow and determines the intra and extracellular *pH* value of neurons (Tombaugh and Somjen, 1997; Bonnet and Wiemann, 1999; Mangieri et al., 2014). The ATPase H+ transporting V1 subunit A (*ATP6V1A*) gene that encodes V-ATPase subunit A, have been reported to be associated with developmental and epileptic encephalopathy 93 (DEE-93, MIM: 618012) (Fassio et al., 2018). However, the association between *ATP6V0C* gene variants and human disease has not been determined. In the present paper, we identified two *ATP6V0C* mutations that co-segregated with the disease in two unrelated families with six individuals affected. The two *ATP6V0C* mutations, including one missense mutation (c.64G > A/p.Ala22Thr) and one frameshift mutation (c.361_373del/p.Thr121Profs*7), were considered to be potentially deleterious because of the possibility of giving rise to alteration of the structure of proteolipid c-ring or producing a truncated transcript. The frequencies of the two *ATP6V0C* mutations in this cohort were statistically higher than that in the control groups of gnomAD and our 296 normal subjects. These data indicated that the *ATP6V0C* mutations were potentially associated with FS or FS related epilepsy.

Previous studies have described 16p13.3 microdeletions in several patients with epilepsy and microcephaly (Mucha et al., 2019; Tinker et al., 2020). The genetic abnormalities involved *ATP6V0C* and other genes such as *AMDHD2, CEMP1, PDPK1*, and *TBC1D24*. Two of the genes were suspected to be pathogenic, including *PDPK1* that was categorized as a highly constrained gene due to the pLI score > 0.95 (Samocha et al., 2014; Feng et al., 2019) and *TBC1D24* that had an established association with epilepsy (Duru et al., 2010; Falace et al., 2010; Luthy et al., 2019). An *ATP6V0C de novo* variant had been reported in a *SCN1A*-negative patient with Dravet syndrome while the patient simultaneously harbored *de novo* missense variants in the genes of *NKAIN3* and *SLC8A1* (Carvill et al., 2014). Recently, a *de novo* stop-loss variant in *ATP6V0C* had been identified in a patient with epilepsy and intellectual disability (Ittiwut et al., 2021). These data provided possible clues for the association between *ATP6V0C* and epilepsy, but the pathogenic role of *ATP6V0C* variants in those cases could not be determined due to the co-appearance of variants in other potentially pathogenic genes or the single affected case. In the present study, we identified *ATP6V0C* mutations in two families with six individuals affected; and the other possible pathogenic genes were excluded. Therefore, this study provided more direct evidence in supporting the association between *ATP6V0C* and epilepsy.

Experiments in zebrafish embryos showed that loss-of-function of *ATP6V0C2* led to a complete inhibition of depolarization-evoked Ca2+ influx in neurons (Chung et al., 2010). Homozygous *ATP6V0C* knockout mice exhibit neonatal lethality, abnormal embryonic tissue morphology, and failure of zygotic cell division (see text footnote 2), suggesting a pathogenic role of loss of function. In the present study, the *ATP6V0C* mutations included one frameshift mutation and one missense mutation. The frameshift mutation resulted in a loss of function by yielding a premature termination of 28 residues at the C-terminus of the protein. The missense mutation was located in the proteolipid c-ring that cooperated with a-subunit forming the hemichannel for proton transferring. It also affected the hydrogen bonds with surround residues and the protein stability, implying a damaging effect. Thus, loss of function was potentially the underlying mechanism of the pathogenicity of *ATP6V0C* mutations. However, the direct functional effects of the *ATP6V0C* mutations were not examined, on which further studies are needed.

All the six affected individuals suffered from their first FS at the age of 7–8 months. The two probands later manifested afebrile seizures including myoclonic seizures that were similar to Dravet syndrome caused by *SCN1A* mutations. However, the patients with *ATP6V0C* mutations displayed good prognosis without psychomotor development disorders, although afebrile seizures or frequent seizures occurred. The myoclonic seizures responded well to LTG, which potentially produce seizure aggravation in patients with *SCN1A* mutations. This study showed a significant difference in clinical prognosis between the epilepsy caused by *ATP6V0C* mutations and Dravet syndrome caused by *SCN1A* mutations. Screening for *ATP6V0C* mutations thus imply clinical significance.

## Conclusion

In conclusion, this study identified *ATP6V0C* mutations in two unrelated families with six individuals affected by FS or EFS+. All affected patients suffered from their first FS at the age of 7–8 months and displayed good prognosis without psychomotor development abnormalities. The frequencies of *ATP6V0C* mutations identified in this cohort were significantly higher than that in the control populations. These findings suggested that *ATP6V0C* is potentially a causative gene in FS or EFS+. Screening for *ATP6V0C* mutations would help differentiating patients with Dravet syndrome, which presented similar clinical manifestation but different responses to antiepileptic treatment.

## Data Availability Statement

The datasets presented in this study can be found in online repositories. The names of the repository/repositories and accession number(s) can be found in the article/Supplementary Material.

## Ethics Statement

The studies involving human participants were reviewed and approved by the Ethics Committee of Guangzhou Women and Children’s Medical Center and the Ethics Committee of Guangdong General Hospital. Written informed consent to participate in this study was provided by the participants’ legal guardian/next of kin. Written informed consent was obtained from the individual(s) and minor(s)’ legal guardian/next of kin, for the publication of any potentially identifiable images or data included in this article.

## Author Contributions

YT, W-PL, and JW designed the study. YT, Q-XZ, X-JL, ZS, CH, W-BL, and W-XC recruited the patients and analyzed the clinical data. C-FC, C-XF, Y-YH, SL, W-PL, and JW analyzed the genetic data. YT, BT, and YZ performed the computational modeling. YT and JW wrote the manuscript. W-PL revised the manuscript. All authors have read and approved the final manuscript.

## Conflict of Interest

The authors declare that the research was conducted in the absence of any commercial or financial relationships that could be construed as a potential conflict of interest.

## Publisher’s Note

All claims expressed in this article are solely those of the authors and do not necessarily represent those of their affiliated organizations, or those of the publisher, the editors and the reviewers. Any product that may be evaluated in this article, or claim that may be made by its manufacturer, is not guaranteed or endorsed by the publisher.

## Footnotes

1
https://www.gtexportal.org/home/

2
http://www.informatics.jax.org/marker/MGI:88116

3
https://www.omim.org/

4
http://varcards.biols.ac.cn/

5
http://www.sbg.bio.ic.ac.uk/phyre2/html/page.cgi?id=index

6
https://alphafold.ebi.ac.uk/entry/P27449

7
http://pymol.org/2/

8
http://gpcr2.biocomp.unibo.it/cgi/predictors/I-Mutant3.0/I-Mutant3.0.cgi

9
https://www.uniprot.org
