# Corrigendum: *ATP6V0C* is associated with febrile seizures and epilepsy with febrile seizures plus

**DOI:** 10.3389/fnmol.2024.1385915

**Published:** 2024-03-01

**Authors:** Yang Tian, Qiong-Xiang Zhai, Xiao-Jing Li, Zhen Shi, Chuan-Fang Cheng, Cui-Xia Fan, Bin Tang, Ying Zhang, Yun-Yan He, Wen-Bin Li, Sheng Luo, Chi Hou, Wen-Xiong Chen, Wei-Ping Liao, Jie Wang

**Affiliations:** ^1^Department of Neurology, Guangzhou Women and Children's Medical Center, Guangzhou Medical University, Guangzhou, China; ^2^Department of Pediatrics, Guangdong Provincial People's Hospital, Guangdong Academy of Medical Sciences, Guangzhou, China; ^3^Department of Neurology, Institute of Neuroscience, The Second Affiliated Hospital of Guangzhou Medical University, Guangzhou, China; ^4^Key Laboratory of Neurogenetics and Channelopathies of Guangdong Province, Ministry of Education of China, Guangzhou, China; ^5^The Seventh Affiliated Hospital, Sun Yat-sen University, Shenzhen, China

**Keywords:** *ATP6V0C*, loss of function, febrile seizures, epilepsy with febrile seizures plus, whole-exome sequencing

In the published article, there was an error in Panel B of Figure 1 as published. On the Case 2 pedigree, the mother's and father's chromatograms have been reversed. The corrected Figure 1 and its caption “Figure 1 | Genetic data of cases with *ATP6V0C* mutations. ” appear below.

**Figure 1:**
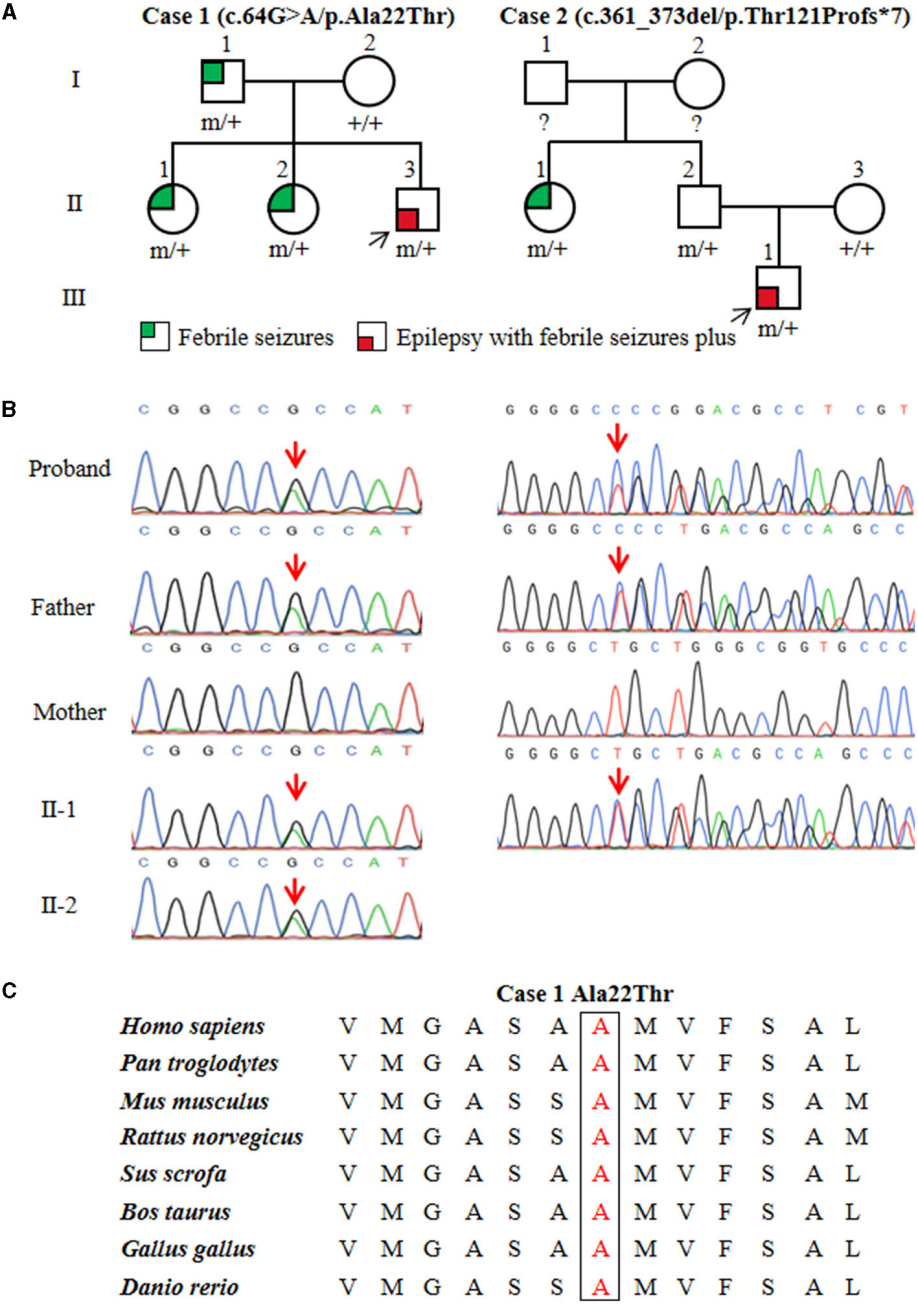
Genetic data of cases with *ATP6V0C* mutations. **(A)** Pedigrees of the two families with *ATP6V0C* mutations and their corresponding phenotypes. Individuals with mutation are marked as m/+, and those without mutation are marked as +/+. **(B)** DNA sequencing chromatograms of the two families with *ATP6V0C* mutations. Red arrows indicate the positions of the mutations. **(C)** Amino acid sequence alignment of the missense mutation shows that residue Ala22 is highly conserved in various species.

The authors apologize for this error and state that this does not change the scientific conclusions of the article in any way. The original article has been updated.

