# Supplementary figures and images for: ATP6V0C Is Associated With Febrile Seizures and Epilepsy With Febrile Seizures Plus
Source: Front Mol Neurosci. 2022 May 6;15:889534. doi: 10.3389/fnmol.2022.889534 (PMC9120599; doi:10.3389/fnmol.2022.889534)

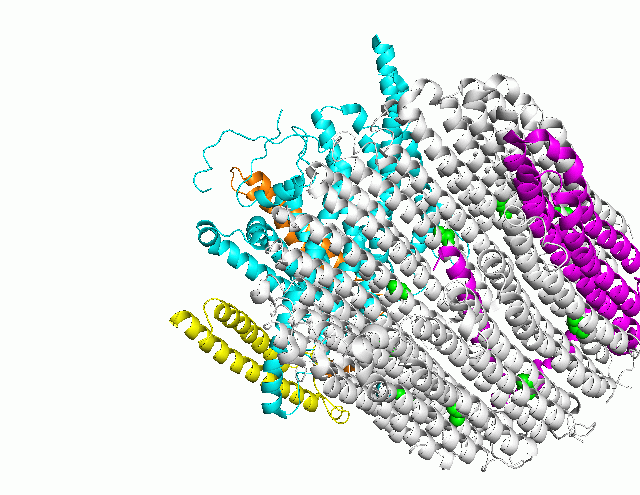

Supplement: Supplementary file 1 [file Data_Sheet_1.ZIP › Tian -Figure s1.gif]
