# Supplementary material for: ATP6V0C Is Associated With Febrile Seizures and Epilepsy With Febrile Seizures Plus
Source: Front Mol Neurosci. 2022 May 6;15:889534. doi: 10.3389/fnmol.2022.889534 (PMC9120599; doi:10.3389/fnmol.2022.889534)
Supplement: Supplementary file 2 [file Table_1.docx]

**Table S1. Genetics characteristics of *ATP6V0C* mutations identified in this study**

|  | **Case 1** | **Case 2** |
| --- | --- | --- |
| cDNA change (NM_001694) | c.64G>A | c.361_373del |
| Protein change | p.Ala22Thr | p.Thr121Profs*7 |
| Inheritance pattern | Segregates with father | Segregates with paternal aunt^§^ |
| MAF | NA | NA |
| MAF-EAS | NA | NA |
| SIFT | Damaging (0.004) | NA |
| PP2_Div | Benign (0.115) | NA |
| PP2_Var | Benign (0.178) | NA |
| LRT | Unknown (0.000) | NA |
| Mutation-Taster | Disease_causing (1) | NA |
| Mutation-Assessor | High (3.77) | NA |
| FATHMM | Tolerable (0.39) | NA |
| PROVEAN | Tolerable (0.43) | NA |
| VEST3 | Damaging (0.725) | NA |
| MetaSVM | Tolerable (-0.342) | NA |
| MetaLR | Tolerable (0.295) | NA |
| M-CAP | Damaging (0.817) | NA |
| CADD | Damaging (26.6) | NA |
| Fathmm-MKL | Damaging (0.947) | NA |
| fitCons | Damaging (0.733) | NA |
| GERP++ | Conserved (3.66) | NA |
| phastCons | Conserved (1.000) | NA |
| phyloP | Conserved (5.300) | NA |
| SiPhy | Conserved (12.965) | NA |
| REVEL | Damaging (0.402) | NA |

^§^The mutation is inherited in an autosomal dominant mode with incomplete penetrance.

Abbreviations: CADD, combined annotation dependent depletion; FATHMM, Functional Analysis through Hidden Markov Models; Fathmm-MKL, Functional Analysis through Hidden Markov Models–Multiple Kernels Learning; fitCons, fitness consequences of functional annotation; GERP, Genomic Evolutionary Rate Profiling; LRT, A likelihood ratio test; MAF, minor allele frequency from Genome Aggregation Database; MAF-EAS, minor allele frequency from Genome Aggregation Database-East Asian population; M-CAP, Mendelian Clinically Applicable Pathogenicity; MetaLR, Meta Logistic Regression; MetaSVM, Meta Support Vector Machine; NA, not available; phastCons, Phylogenetic Analysis with Space/Time models conservation scoring and identification of conserved elements; phyloP, Phylogenetic Analysis with Space/Time models Computation of p-values for conservation or acceleration, either lineage-specific or across all branches; PP2_Div, polyphen2_HDIV; PP2_Var, polyphen2_HVAR; PROVEAN, Protein Variation Effect Analyzer; REVEL, Rare Exome Variant Ensemble Learner; SIFT, Sorting Intolerant From Tolerant; SiPhy, Site-specific PHYlogenetic analysis; VEST3, The Variant Effect Scoring Tool 3.0.
